# Susceptibility of Human Embryonic Stem Cell-Derived Neural Cells to Japanese Encephalitis Virus Infection

**DOI:** 10.1371/journal.pone.0114990

**Published:** 2014-12-17

**Authors:** Shih-Cheng Shen, Ching-I Shen, Ho Lin, Chun-Jung Chen, Chia-Yu Chang, Sheng-Mei Chen, Hsiu-Chin Lee, Ping-Shan Lai, Hong-Lin Su

**Affiliations:** 1 Department of Life Sciences, Agricultural Biotechnology Center, National Chung Hsing University, Taichung, Taiwan; 2 Department of Chemistry, National Chung Hsing University, Taichung, Taiwan; 3 Department of Education and Research, Taichung Veterans General Hospital, Taichung, Taiwan; 4 Center for General Education, Tunghai University, Taichung, Taiwan; 5 Graduate School of Nursing, Hung-Kuang University, Taichung, Taiwan; 6 Ph.D. Program in Tissue Engineering and Regenerative Medicine, National Chung Hsing University, Taichung, Taiwan; Utah State University, United States of America

## Abstract

Pluripotent human embryonic stem cells (hESCs) can be efficiently directed to become immature neuroepithelial precursor cells (NPCs) and functional mature neural cells, including neurotransmitter-secreting neurons and glial cells. Investigating the susceptibility of these hESCs-derived neural cells to neurotrophic viruses, such as Japanese encephalitis virus (JEV), provides insight into the viral cell tropism in the infected human brain. We demonstrate that hESC-derived NPCs are highly vulnerable to JEV infection at a low multiplicity of infection (MOI). In addition, glial fibrillary acid protein (GFAP)-expressing glial cells are also susceptible to JEV infection. In contrast, only a few mature neurons were infected at MOI 10 or higher on the third day post-infection. In addition, functional neurotransmitter-secreting neurons are also resistant to JEV infection at high MOI. Moreover, we discover that vimentin intermediate filament, reported as a putative neurovirulent JEV receptor, is highly expressed in NPCs and glial cells, but not mature neurons. These results indicate that the expression of vimentin in neural cells correlates to the cell tropism of JEV. Finally, we further demonstrate that membranous vimentin is necessary for the susceptibility of hESC-derived NPCs to JEV infection.

## Introduction

Japanese encephalitis virus (JEV), which belongs to the flavivirus family and contains a positive-sense, single-stranded RNA genome, is a severe public-health threat in Asia [1]. Patients infected with Japanese encephalitis virus (JEV) who do not obtain the proper vaccination or treatment may develop acute encephalitis and have a high mortality rate [2]. Neuropathological features found in JEV-infected brains include multiple foci of acellular necrotic plaques in gray matter areas, such as the cerebral cortex, hippocampus, thalamus, substantia nigra and medulla oblongata [3], [4], [5], [6]. Reactivated astrocytes and microglia nodules aggregate in the surrounding damaged inflammatory regions, which are accompanied by edema, hemorrhage and extensive perivascular inflammatory infiltrates [3], [7]. Neuronal cells, such as the pyramidal neurons in the hippocampus and spinal cord, have been reported as the primary target cells of JEV [2], [7]. Immunohistological observations reveal that the viral antigens can also be detected in astrocytes, microglia, vascular endothelial cells and ependymal cells [7].

Although immunohistochemical studies indicate the correlation of Japanese encephalitis and severe neuron loss, direct evidence is still lacking as to whether primary viral infection or a secondary immunological cytokine storm causes the death of neurons. In addition, the cell identification of the most reported JEV tropism in autopsied brains or primary cultures relies on the morphological features of infected cells [6], [7], [8], [9]. Further confirmation is required by using human neural cells for JEV primary infection and applying immunological double-staining with both viral antigens and cell lineage-specific markers.

Here we used human embryonic stem cell (hESC)-derived neuroepithelial precursor cells (NPCs) and functional mature neural cells to investigate the cell tropism of JEV infection. The hESC-derived specific NPCs and mature neural cells can be sequentially generated as embryonic developmental stages, and the derived neurons faithfully recapitulate the same neurophysiological properties as ex vivo neuronal cells [10], [11], [12]. Here, we carefully evaluate the infectivity and cell tropism of JEV in early-stage NPCs and late-stage mature neural cells by using a Taiwan-isolated neurovirulent JEV strain [13]. Our results show that NPCs and glial cells, but not mature neurons, are the primary targeted cells for JEV infection in humans.

## Materials and Methods

### Viruses and titer determination

A plaque-purified neurovirulent RP-9 JEV strain [13] was a gift from Dr. Yi-Ling Lin at Academia Sinica Taiwan and amplified in mosquito C6/36 cells, which were cultured in RPMI 1640 medium with 5% fetal bovine serum (FBS, Invitrogen, Carlsbad, CA, USA). Baby hamster kidney fibroblast cells (BHK-21) were grown in RPMI 1640 medium containing 5% FBS and 2 mM L-glutamine and used for the viral plaque assay. JEV titer was determined by the number of JEV plaques in infected BHK-21 cells on fourth day post-infection (4 d.p.i.), revealed by crystal violet staining.

### hESC cultures

The TW1 hESC lines (XY, passages 80–90) were grown in mTeSR1 media (Stem Cell Technologies, Vancouver, BC, Canada) on 1% Matrigel (Becton Dickinson, BD, Franklin Lakes, NJ, USA) coated 6 cm dishes (Corning, Corning, NY, USA). The TW1 cells have been previously described [14] and were obtained from Lee Women's Hospital in Taiwan. Following the Policy Instructions on the Ethics of Human Embryo and Embryonic Stem Cell Research, the Institutional Review Board of the Industrial Technology Research Institute, Hsinchu, Taiwan approved the establishment of hESC lines from surplus blastocysts donated by Taiwanese infertile couples undergoing IVF treatment at Lee Women's Hospital, Taichung, Taiwan. Both parties of each couple signed an informed- consent form after receiving oral and written information about the research [14]. The culture medium for the hESCs was refreshed daily. The cells were passaged using dispase II (0.5 mg/ml, Invitrogen) and replated at a cell dilution of 1∶5 to 1∶8.

### Neural induction

After being treated with dispase II for 5 min, the detached hESCs (1.0×10^6^ cells) were partially dissociated into 200–300 µm cell clusters and transferred to 6 cm bacterial-culture dishes (Alpha Plus, Taiwan) for 2 days of culturing. The differentiating medium contained DMEM-F12 (Invitrogen) and 20% knockout serum replacement (KSR, Invitrogen), 1 mM non-essential amino acids (NEAAs, Invitrogen), 2 mM glutamate (Invitrogen) and 0.1 mM 2-mercaptoethanol (Invitrogen). The day of cell dissociation was set as D1. From D3 to D4, the cell suspensions were cultured in DMEM-F12 with 1% N2 supplement (Invitrogen), 1 mM NEAAs and 2 mM glutamate. Neural inducing factors were applied during this D3–D4 stage, including 10 µM SB431542 (Sigma-Aldrich, St. Louis, MO, USA), 0.5 µM BIO (Sigma-Aldrich) and 10 ng/mL recombinant human FGF-2 (rh-FGF2, R&D Systems, Minneapolis, MN, USA). From D5, the neural inducing factors were removed, and the cells were further cultured in neurobasal media (Invitrogen) with 1% N2 supplement and 10 ng/mL rh-FGF2.

### Production of different subtypes of neurons

The glutamate and γ-aminobutyric acid (GABA)-secreting neurons were autonomously differentiated from the NPCs in neurobasal medium with N2 and B27 supplements (Invitrogen). The procedure for serotonergic neuron and dopaminergic neuron induction was modified from previous reports [11], [15], [16]. Briefly, the NPCs were adhered to 1% Matrigel-coated plates on D10. The formation of dopaminergic neurons was induced by the addition of 40 µg/mL FGF8b (R&D Systems) and 1 µM purmorphamine (Merck-Millipore, Billerica, MA, USA), a sonic hedgehog (Shh) signal activator [17], from D11 to D15. The medium was refreshed every 2 days. To promote terminal neural differentiation, a cocktail containing 10 ng/mL brain-derived neurotrophic factor (BDNF, Peprotech, Rocky Hill, NJ, USA), 10 ng/mL glia cell line-derived neurotrophic factor (GDNF, Peprotech) and 5% B27 supplement (Invitrogen) in neurobasal media was provided from D16 [18]. Serotonergic neurons and dopaminergic neurons were examined by immunocytostaining on D25.

### Immunocytochemistry (ICC) staining

Cells were fixed with 4% paraformaldehyde (Sigma-Aldrich) for 5 min and washed twice with phosphate buffered saline (PBS, Sigma-Aldrich). After membrane permeabilization with 0.3% Triton-100 (USB, Cleveland, OH, USA), cellular antigens were first blocked with 5% horse serum (Invitrogen) and then stained with primary antibodies at 4°C overnight. The primary antibodies used in this study included Oct4 (1∶500; Santa Cruz Biotechnology, Dallas, TA, USA), Sox1 (1∶200, Santa Cruz Biotechnology), Pax6 (1∶100, Covance, Princeton, NJ, USA), nestin (1∶500, Covance), βIII-tubulin (TuJ1, 1∶500, Covance), glial fibrillary acidic protein (GFAP, 1∶500, Millipore), GABA (1∶500, Millipore-Chemicon), glutamate (1∶500, Sigma-Aldrich), tyrosine hydroxylase (TH, 1∶100, Covance) and serotonin (1∶1000, Sigma-Aldrich). JEV infection was determined by an anti-JEV NS1 monoclonal antibody, a gift from Dr. Yi-Ling Lin at Academia Sinica in Taiwan. Cell nuclei were stained with diamidino-2-phenylindole (DAPI, Invitrogen). Fluorescent cell images were obtained using an upright microscope (Eclipse TE2000-S and 80i, Nikon, Tokyo, Japan) or a confocal microscope (LSM 510, Carl Zeiss, Oberkochen, Germany). The population ratios of specific protein expressing cells were estimated by manual counting with AlphaEase FC software (Alpha Innotech, San Leandro, CA, USA).

### Western immunoblot analysis

Cells were dissolved in sodium dodecyl sulfate (SDS) sample buffer (62.5 mM Tris-HCl, pH 6.8, 2% SDS, and 10% glycerol) containing a protease inhibitor cocktail (Sigma-Aldrich). Total proteins were separated by SDS-polyacrylamide gel electrophoresis (SDS-PAGE) and transferred to a PVDF membrane (GE Life Science-Amersham, Pittsburgh, PA, USA). The membranes were blocked with 5% horse serum and then incubated with the anti-JEV NS1 monoclonal antibody. A horseradish peroxidase-conjugated secondary antibody (Jackson ImmunoResearch, West Grove, PA, USA) was subsequently added, and the blots were developed with ECL reagents (GE Life Science-Amersham).

### Infection inhibition by anti-vimentin antibodies (Abs)

The viral inhibition assay is following a previous report [21]. Briefly, the differentiating NPCs (10^5^ cells) were grown on 12-well plates and treated with a 50 fold diluted preimmune Ab (Sigma-Aldrich, I9140) or goat anti-vimentin polyclonal antibody (Sigma-Aldrich, V4630, 1∶50 or 1∶100) for 1 h at 4°C. The cells were infected JEV RP-9 strain at a MOI of 0.2 in the presence of antibodies for 2 h at 4°C. The unbound viruses were washed out with cold serum-free RPMI and further incubated for 12 or 24 h at 37°C. The ratios of JEV-infected cells were revealed by the expression of JEV NS1 protein and estimated by manual counting and a densitometric software (AlphaEase FC). The cultured supernatants were collected for virus titration by plaque formation assay.

### Statistical analysis

Representative data were collected from at least two independent experimental results and shown as mean value±standard deviation. Statistical analyses were conducted using one-way ANOVA and the significance was examined by Tukey's post-hoc assay.

## Results

### Robust neural formation of TW1 hESCs

**Figure 1:**
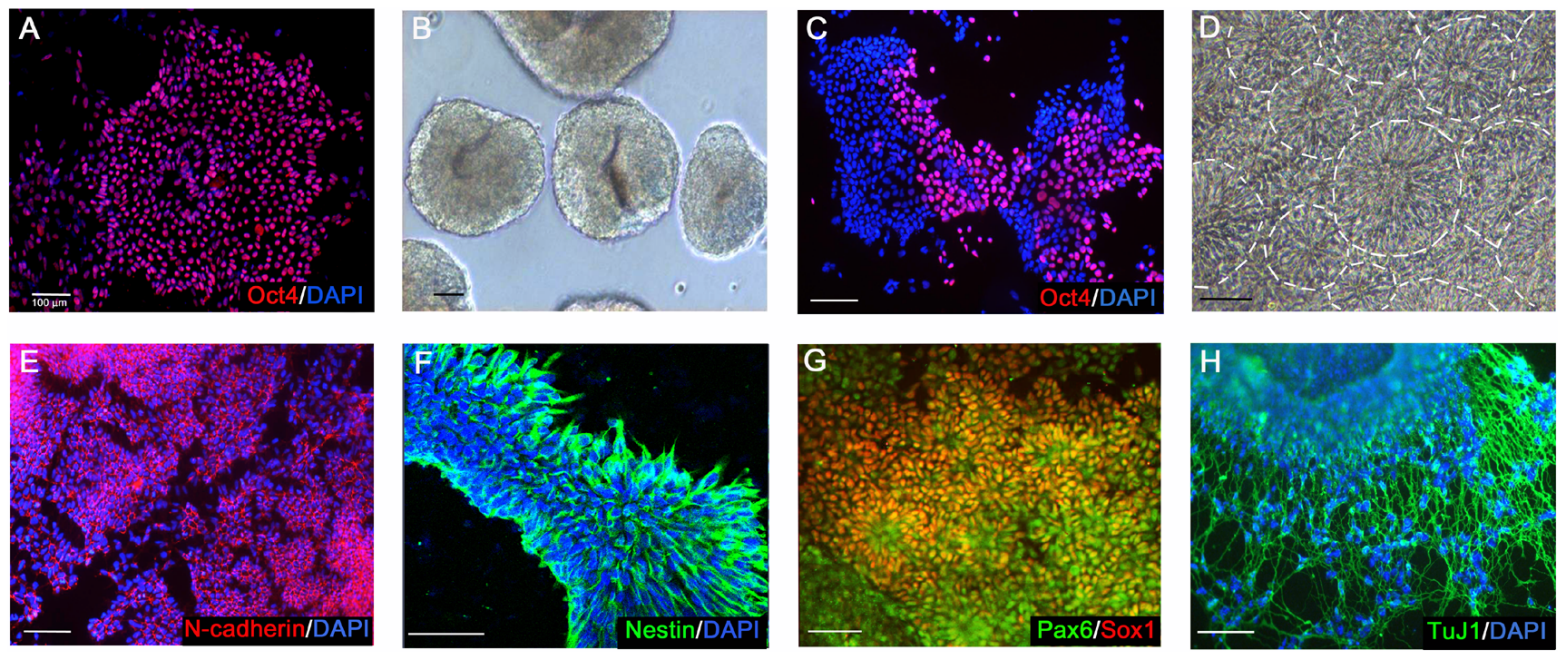
Efficient neural differentiation of TW1 hESCs. (A) The Oct4 transcription factor, a well-characterized pluripotent marker, was detected in undifferentiated TW1 hESCs. (B) Dissociated hESCs aggregated in suspension and formed numerous embryonic bodies. (C, D) Differentiating hESCs exhibited reduced expression of Oct4 on D5 (C) and displayed a classical neural rosette conformation after plating on culture plates on D10 (D). (E–G) NPCs express N-cadherin (E), nestin (F), Pax6 and Sox1 transcription factors (G). (H) Extensive TuJ1^+^ neurites were detected in NPC-derived mature neurons on D25. Scale bars, 100 µm.

Undifferentiated TW1 hESCs, expressing the pluripotent Oct4 gene (Fig. 1A), were propagated in non-serum, non-feeder and chemically defined mTeSR1 medium. After cell dissociation with dispase, suspended dissociated TW1 hESCs formed embryoid bodies in DMEM-F12 media containing 20% knock-out replacement serum (KSR) (Fig. 1B). The cells were directed toward neural differentiation by transient treatment with 0.5 µM BIO/10 µM SB431542/10 ng/mL FGF2 (BiSF) on D3 for 2 days and further cultured with 10 ng/mL FGF2 in neurobasal medium with N2 supplement from D5 [12]. The initial neural differentiation was accompanied by a dramatic reduction in Oct4 expression (38.4±4.7%) on D5 (Fig. 1C). On D10, over 90% of differentiating hESCs consistently displayed a classical neural rosette conformation (marked with circular margin lines) after plating on culture plates (Fig. 1D), and the neural cell lineages were further confirmed by the expression of specific NPC markers, including N-cadherin (Fig. 1E), nestin (Fig. 1F), Pax6 and Sox1 transcription factors (Fig. 1G) [11]. Robust neurite formation of the neurospheres was observed on D25, characterized by the expression of the neuronal-specific βIII tubulin protein (stained with TuJ1 antibody, Fig. 1H). We also noticed that less than 5% of differentiating hESCs expressed Sox17 or Brachyury genes, the specific markers of endoderm and mesoderm respectively, indicating the high purity of the neural population and low contamination with endomesodermal cells in our neural culture system [12].

### JEV infection in hESCs and hESC-derived NPCs

**Figure 2:**
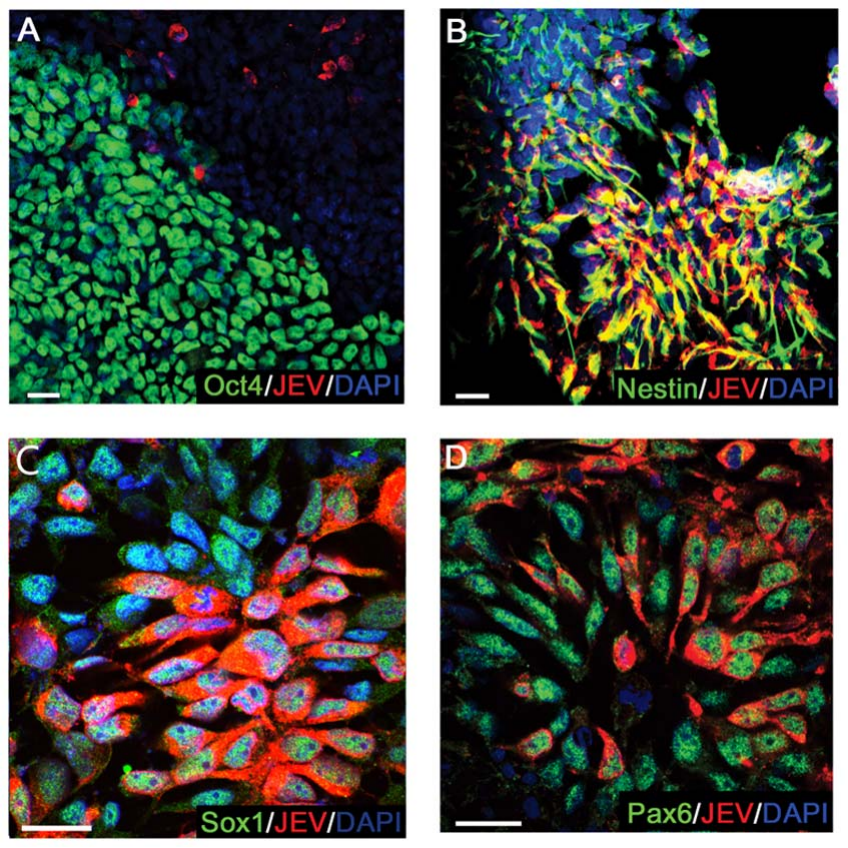
JEV infection in hESC-derived NPCs. (A) Infection by JEV, detected by an anti-JEV NS1 monoclonal antibody, was observed in Oct4 null cells of differentiating hESCs at MOI 1. (B–D) Infection by JEV in NPCs was determined by detecting the viral antigen in nestin (B), Sox1 (C) and Pax6 (D) expressing cells. Scale bars, 50 µm.

Differentiating hESCs on D5, containing both undifferentiating Oct4^+^ cells and NPCs, were infected with JEV at MOI 1 at 1 d.p.i.. We found that JEV NS1 proteins were not detected in pluripotent Oct4^+^ hESCs (Fig. 2A). This result is similar to a previous study of Western Nile virus in mouse ESCs [19]. In contrast, the viral NS1 protein was detected in Nestin/Sox1/Pax6 positive cells at MOI 1 at 1 d.p.i., illustrating that NPCs are susceptible to JEV infection (Fig. 2B–D). This result indicates that hESC-derived NPCs, but not the undifferentiated hESCs, are susceptible to JEV infection.

**Figure 3:**
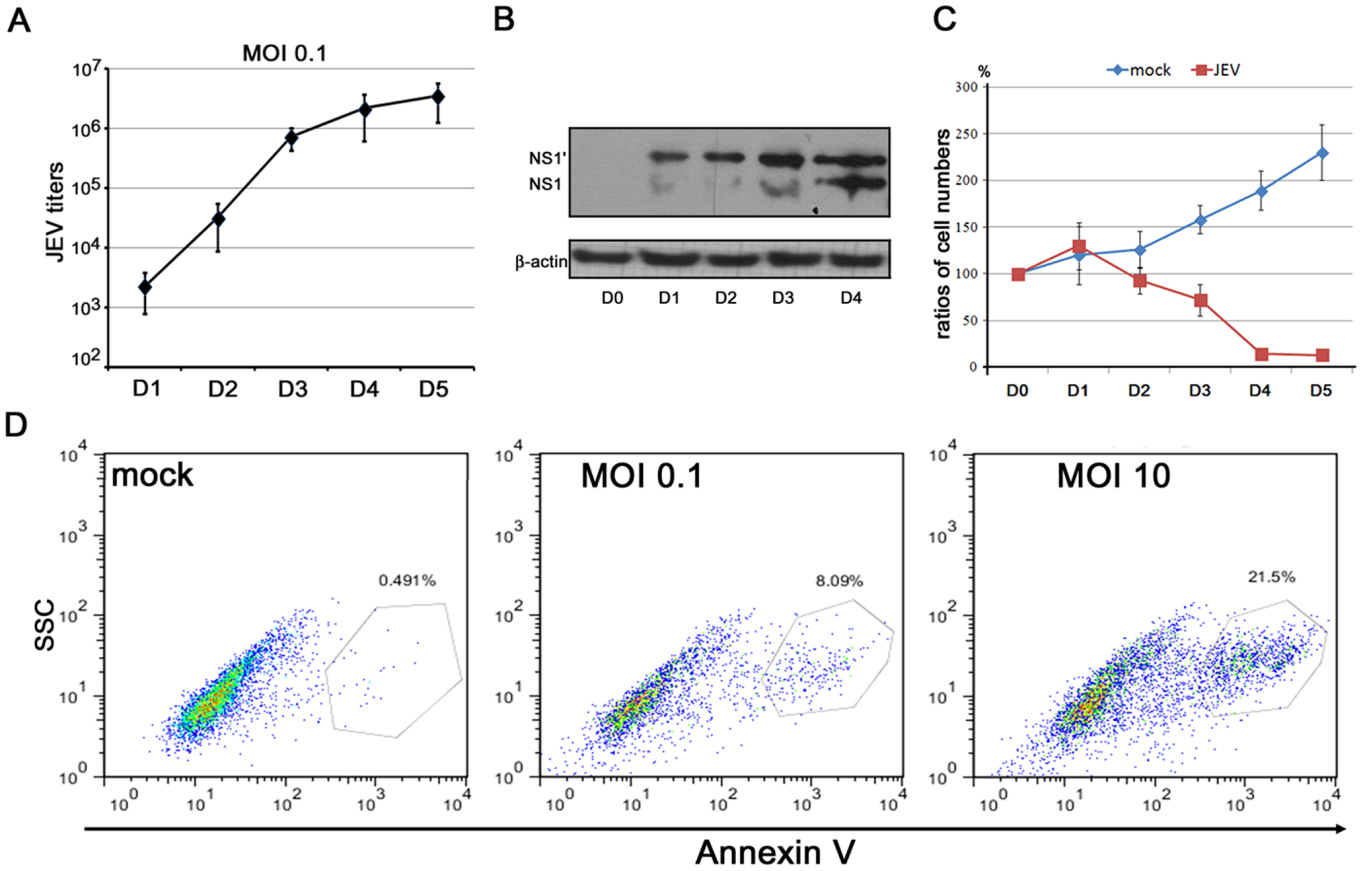
Viral replication and cytopathic effect of JEV-infected NPCs. (A) The viral growth curve in JEV-infected NPCs at MOI 0.1 is illustrated. (B, C) The infected NPCs expressed the viral NS1 and NS1′ proteins (B) and exhibited a depleted cell population (C) at MOI 0.1. (D) The degree of membrane externalization on the infected cells, detected by Annexin V staining, was quantified by flow cytometry analysis.

NPCs on D10 also supported the viral growth at MOI 0.1 and produced extracellular infectious JEV virions after 3 d.p.i. (Fig. 3A). The viral growth kinetics were correlated to the intracellular production of viral NS1 and NS1′ proteins (Fig. 3B). Massive cell death of the infected NPCs was observed on 3 d.p.i. (72±17% survival of original cells) and 4 d.p.i. (14.6±6.5% survival of original cells) at MOI 0.1 (Fig. 3C). The ratios of membrane externalization of phosphotidylserine, detected by FITC-Annexin V and flow cytometry, revealed that 8% and 22% NPCs underwent apoptosis at 24 h.p.i. at MOI 0.1 and 10, respectively (Fig. 3D).

### JEV infection in hESC-derived mature neural cells

**Figure 4:**
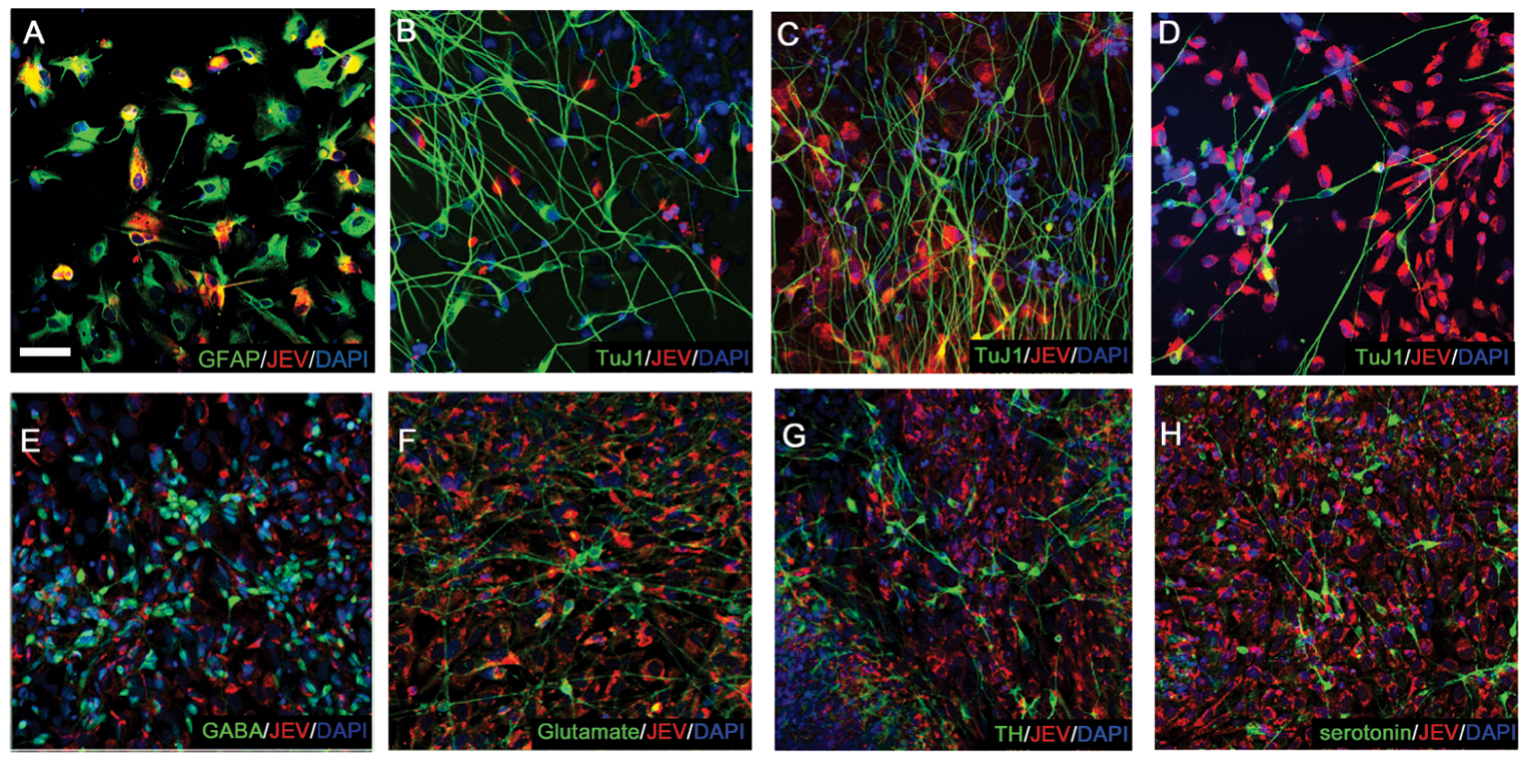
JEV infection in hESC-derived glial cells and neurons. (A) The JEV NS1 proteins were detected in GFAP-expressing glial cells at MOI 1 on 1 d.p.i.. (B–D) The expression of JEV NS1 proteins in mature neurons (TuJ1^+^) is illustrated at MOI 1 on 1 d.p.i. (B) and at MOI 10 on 1 d.p.i. (C) or 3 d.p.i. (D). (E–H) The infectivity of JEV was determined in GABAnergic (E), glutaminergic (F), dopaminergic (G) and serotonergic neurons (H) at MOI 10 on 3 d.p.i. TuJ1 antibody, anti-βIII tubulin. TH, tyrosine hydroxylase. Scale bar, 20 µm.

To examine the JEV infection profile in human mature neural cells, several neurotrophic factors, such as GDNF and BDNF, and B27 supplements were added to promote the terminal differentiation of the TW1 hESCs-derived NPCs [12]. Radial glia and astrocytes were both identified by the expression of GFAP, whereas the neurite-bearing neurons were characterized by the presence of β-III tubulin expression (TuJ1^+^) [20]. We discovered that GFAP^+^ glial cells (53±14% of GFAP^+^ cells, n = 742) (Fig. 4A) were much more susceptible than TuJ1^+^ neurons (1.4±0.6%, n = 711) (Fig. 4B) to JEV infection at MOI 1. No obvious cell death or enhanced cell proliferation of GFAP^+^ cells was observed after JEV infection at 3 d.p.i..

To further investigate JEV infection in mature neurons, differentiating neural cells on D25 were incubated with virus at MOI 10 for 1 day (Fig. 4C) or 3 days (Fig. 4D) without media replacement. Due to the low contamination of non-neural lineages within the ESC-derived population (Fig. 1), most of the JEV-infected cells showing non-spindle-shaped morphology (red cells in Fig. 4B–H) may represent the NPCs or GFAP^+^ glial cells. Examining the neurite-extending cells with the βIII tubulin expression (TuJ1^+^, green cells in Fig. 4C and 4D), we found that only 2.6±1.2% (n = 824) (Fig. 4C) and 8.5±2.1% (n = 705) (Fig. 4D) of TuJ1^+^ neurons expressed JEV NS1 proteins in the cytosol at 1 d.p.i. and 3 d.p.i., respectively. We also found that functional neurotransmitter-secreting neurons, including GABAnergic (GABA^+^, Fig. 4E), glutaminergic (glutamate^+^, Fig. 4F), dopaminergic (TH^+^, Fig. 4G) and serotonergic neurons (serotonin^+^, Fig. 4H), almost lacked JEV NS1 expression (red signals in Fig. 4) at MOI 10 at 3 d.p.i.. These results clearly illustrate that mature neuronal cells, even grown with JEV-infected NPCs and glial cells and infected with high MOI, are generally resistant to JEV infection.

### Cell tropism of JEV infection correlates to the vimentin expression

**Figure 5:**
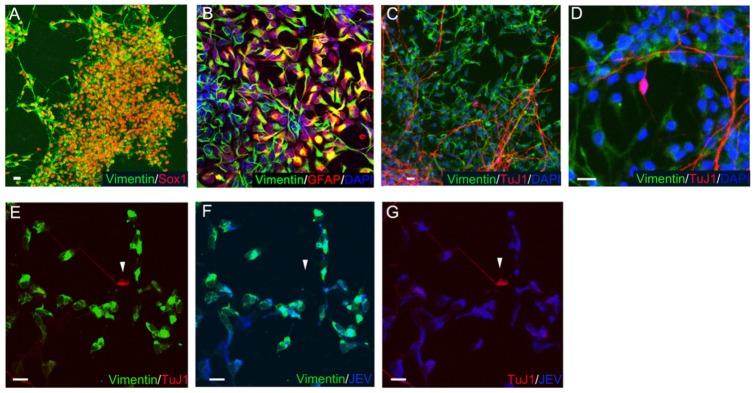
JEV infection in vimentin-expressing cells. (A, B) Vimentin expression was observed in most Sox1^+^ NPC cells and GFAP^+^ cells. (C, D) Most TuJ1^+^ expressing cells (matured neurons) did not express the vimentin. Panel C and D, low magnification and high magnification, respectively. (E, F, G) JEV-infected cells (JEV NS1^+^ cells, blue) were colocalized with the vimentin-expressing cells (green), but not the human mature neurons (TuJ1^+^, arrow head, red). Scar bar in each panel, 10 µm.

A recent study of JEV indicated that the intermediate filament vimentin on cell membrane mediates viral entry and neural infectivity and determines the viral virulence in central nerve system (CNS) of mice [21]. To evaluate the role of vimentin in JEV-infected human neural cells, we first examined the expression profile of vimentin in hESC-derived NPCs and mature neurons. ICC staining revealed that vimentin was strongly expressed in most Sox1^+^ NPCs (Fig. 5A) and GFAP^+^ glial cells (Fig. 5B), but not mature neurons (TuJ1^+^) (Fig. 5C, D). Infection with JEV did not enhance the vimentin expression in mature neurons (Fig. 5E). Interestingly, we also found that the infectivity of JEV was detected in vimentin-expressing cells (Fig. 5F), whereas most neurite-bearing neurons (TuJ1^+^, arrow head) express a low level of vimentin and were resistant to JEV infection (Fig. 5G) at MOI 10 at 3 d.p.i. (same field for Fig. 5E, 5F, 5G). These observations indicate that the cell tropism of JEV for human neural cells is highly correlated to their vimentin expression.

### Vimentin is a determining factor for the cell tropism

**Figure 6:**
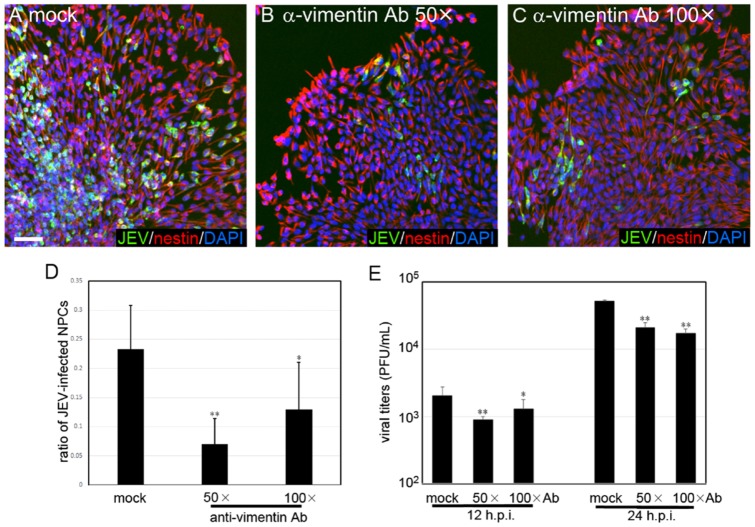
JEV infection in anti-vimentin pretreated NPCs. (A, B, C) Nestin^+^-NPCs (red) were pretreated with preimmune Abs (mock) (A), 1∶50 diluted (B) and 1∶100 diluted (C) anti-vimentin Abs for 1h at 4°C and then were infected with JEV at MOI 0.2 for 1h. The infected NPCs were fixed and stained at 24 h.p.i.. Scale bar, 50 µm. (D) The ratio of JEV-infected cells (expressing viral NS1 protein, green) were counted with the assistance of a densitometric software (total cells>5000, duplicated) (*, p<0.05; **, p<0.01, one-way ANOVA). (E) The released viral particles, collected from the supernatants of infected cells at 12 and 24 h.p.i., were titrated by plaque forming assay (*, p<0.05; **, p<0.01, one-way ANOVA). Representative data were collected from two independent experiments.

To determine the role of vimentin for JEV infection on hESC-derived neural cells, differentiating NPCs on D10 were treated with anti-vimentin antibody at 4°C for 1h and consequently infected with JEV at MOI 0.2. Our results showed that pretreating with anti-vimentin Abs dose-dependently attenuated the ratio of JEV-infected cells at 24 h.p.i., indicating that membranous vimentin is necessary for JEV adsorption (Fig. 6A-D) (**, *p*<0.01, *, p<0.05 in Fig. 6D). In addition, the released infectious viral particles in the media were also significantly reduced in anti-vimentin Ab-treated group, comparing to the control of preimmune Ab-treated cells at 12 and 24 h.p.i. (Fig. 6E). These data suggest that vimentin on outer cell membrane is one of the determining factor for the human neural cell tropism of JEV, at least for the viral susceptibility of NPCs.

## Discussion

The distribution of viral receptors and the intrinsic supporting machinery for viral replication determine the cell tropism of viral infection. In general, the initial receptor binding on the cell membrane is the primary determinant for the specificity of the targeted cell. Both membranous vimentin and highly sulfated glycosaminoglycans (GAGs) have been demonstrated to participate in the entry of JEV into neural cell lines [21], [22]. A recent study of a neurovirulent JEV RP-9 strain indicated that the intermediate filament vimentin on cell membrane interacts with the viral envelope protein and mediates viral entry and neural infectivity, whereas the attenuated JEV RP-2ms primarily uses sulfated GAGs for viral binding to targeted cells [21].

The expression of vimentin in human and rodent brains has been detected in embryonic primitive neuroepithelial cells and radial glia in the developing neural tube [23], [24], [25]. In adult CNS tissues, vimentin expression is observed in ependymal cells and GFAP-positive astrocytes but not in neurons and oligodendrocytes [20], [25], [26]. Interestingly, this expression profile of vimentin faithfully reflects our findings of the neural tropism of JEV, showing that NPCs and glial cells, but not mature neurons, are susceptible to JEV infection. Although the necessity of initial JEV-vimentin binding and consequent internalization of the JEV-vimentin complex for the CNS infection still need validation using RNAi knockdown and vimentin-knockout mice [27], cellular distribution of vimentin in the CNS shed light on the cell tropism of JEV.

In addition to the vimentin expression profile, the age-dependent property of JEV-triggered neuropathology also supports the JEV vulnerability to NPCs in humans [2], [9], [28], [29]. Epidemiological studies indicate that young children are much more susceptible than adults to JEV-triggered brain lesions [2], [28]. Experimental evidence also shows that only neonatal rats, but not the rats older than 2 weeks, succumb to the intracerebral injection of JEV [29]. Immunocytostaining of JEV-infected rodent brains further revealed that immature NPCs and radial glial cells are the primary targeted cell population [30], and infected cells may undergo cell growth arrest [31], resulting in a diminished NPC pool in the infected survivors. Here we first demonstrated that human NPCs are highly susceptible to JEV infection and can support efficient viral proliferation at low MOI. Apoptotic cell death was accompanied by featured outer membrane externalization and high viral production from 3 d.p.i.. These JEV results exhibit similar infection profile to the coxsackievirus [32], [33] but different to the mature-neuron tropism of Varicella-zoster virus on hESC-derived cells [34], [35].

Here we argue that NPCs and glial cells, but not mature neurons, are the primary targeted cells for JEV infection in humans. Regarding to the primary infection of JEV, initial circulating viruses may first invade the blood-brain barrier and infect the endothelial cells and glial cells [8], [40]. Although most mature neurons are resistant to the JEV infection, the cells still could be infected by JEV at high MOI and long-term challenge (Fig. 4D). The infected glial cells may provide a large amount of amplified JEV to attack neighboring mature neurons or neural stem cells and cause the loss of neurons in vivo. In addition, adult neural stem cells, which are located near the endothelial cells, may be persistently infected and propagate the JEV to their progeny of derived neurons [25], [31]. Infection of mature neurons in the late stage of JEV encephalitis may also be triggered by the released cytokines or pro-inflammatory mediators from infected glial cells and activated microglia, which disturb the membrane integrity of surrounding neurons and may consequently facilitate the viral infection and spreading of the cells. Furthermore, infected glial cells may upregulate the expression of JEV co-receptors, such as heparin or haparan sulfate [22], and promote the JEV adsorption and infection to their neighboring neurons. These raised possibilities will be validated to elucidate the detailed mechanisms for the neuronal infection and neuronal cell death in patients.

The finding of viral tropism for the neural cells also emphasizes that attenuating the viral load in adult stem cells and glial cells is critical for the JEV treatment. Alternatively, controlling the cytokine storm produced by the infected glial cells or activated microglia could also partially rescue the survival of neurons after JEV infection [36]. Constitutively activated microglia and their secreted cytokines in JEV-infected brain could exaggerate neuroinflammation and consequently trigger severe neuronal cell death [37], [38], [39]. In vitro studies of antibody neutralization further illustrate that TNF-α and IL-1β released by activated microglia are critical for JEV-induced neurotoxicity [40], [41], [42]. Attenuating the inflammatory response by chemical compounds, such as minocycline, can enhance neurogenesis and diminish the viral pool in the subventricular zone of JEV-inoculated mice [43]. These observations suggest that immune-modulation therapy for preventing cytokine storm and microgliosis may provide neuroprotection and rescue the survival of neuronal cell for JEV-triggered acute encephalitis.

Taken together, we demonstrate that the neural differentiation system of hESCs is useful to investigate the cell tropism and cytopathogical mechanism of human neurovirulent viruses. Our study indicates that NPCs and glial cells, but not the mature neurons, are the primary target cells of JEV. Further investigations of the role of vimentin and cytopathogenesis in JEV-infected NPCs will improve our understanding of the viral-cellular interactions and may help us to design effective treatments for acute viral encephalitis and persistent infection in the infected patients.
